# Reversible Thickness Engineering in Amorphous In_2_O_3_ Transistors

**DOI:** 10.1021/acs.nanolett.5c06228

**Published:** 2026-04-13

**Authors:** Yi-Yu Pan, Chu-Hsiu Hsu, Robert Tseng, Sung-Tsun Wang, Yu-Cheng Chang, Shih-Chieh Chen, Takashi Kimura, Yann-Wen Lan, Der-Hsien Lien

**Affiliations:** † Institute of Electronics, 34914 National Yang Ming Chiao Tung University, Hsinchu 300093, Taiwan; ‡ Institute of Pioneer Semiconductor Innovation, National Yang Ming Chiao Tung University, Hsinchu 300093, Taiwan; § Department of Physics, Kyushu University, 744 Motooka, Fukuoka 819-0395, Japan; ∥ Department of Physics, 596277 National Taiwan Normal University, Taipei 116077, Taiwan

**Keywords:** oxide semiconductors, In_2_O_3_, top-down process, bottom-up process, thickness control, threshold voltage, doping-free

## Abstract

Amorphous oxide semiconductors offer a unique platform for versatile device processing, as their noncrystalline nature eliminates the requirement for lattice matching at the interface and enables low-temperature regrowth and isotropic processing. Leveraging chemical continuity of amorphous surface, this study demonstrates atomic-scale, reversible thickness control of amorphous In_2_O_3_ by integrating bottom-up atomic layer deposition with top-down hydroxide-assisted wet-etching processes. The processes achieve bidirectional modulation of the film thickness from 1 to 4 nm under back-end-of-line compatible conditions while maintaining smooth surfaces (*R*_a_ = ∼0.5 nm) and stable chemical composition. This thinning–regrowth process enables precise, thickness-dependent control over the In_2_O_3_ transistor performance, facilitating doping-free modulation of the threshold voltage and reversible switching between depletion- and enhancement-mode operation within a single device. Successful demonstration of inverters and ring oscillators confirms the robustness of this technique, establishing film thickness as a core design parameter for oxide semiconductors and paving the way for reconfigurable electronics.

## Graphical abstract

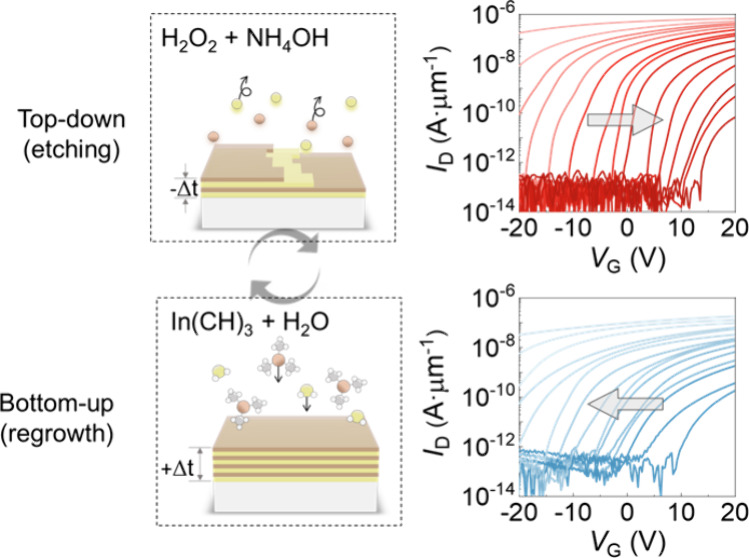

Semiconductor fabrication relies on the coordinated and repeated synergy of bottom-up and top-down processing to integrate materials and structures across multiple layers to develop complex integrated circuits.^1–4^ Bottom-up processes, such as sputtering^5,6^ and atomic layer deposition (ALD),^7,8^ are used to form functional material layers with controlled composition and thickness. Complementarily, top-down processes,^9^ such as plasma etching,^10^ shape these layers into active device architectures with defined geometry. Metals,^11^ dielectrics,^12^ and semiconductors^13^ can, in principle, all be processed by both bottom-up and top-down approaches. Among them, semiconductors impose stricter requirements and are less tolerant of processing variations. For bottom-up growth of semiconductor materials, achieving high crystallinity is essential, as it directly determines carrier mobility and device reliability.^14^ However, high-quality epitaxy usually requires atomically ordered substrates and high temperatures to promote surface diffusion and crystal formation,^15,16^ yet such conditions often cause strain or interdiffusion (e.g., in heterostructures like GaN on Si),^17^ limiting process compatibility. Top-down processes, on the other hand, demand precise control of etching rate and selectivity while maintaining surface morphology and chemical stability, especially in nanoscale devices that are highly sensitive to surface conditions.^10,18^ Conventional etching, however, often alters surface composition and chemical potential, modifying surface energetics and hindering subsequent epitaxial regrowth.^19^

To overcome these constraints, innovative material platforms that decouple processing from strict crystallographic requirements are essential. Two-dimensional (2D) materials are unique material systems in this regard. Their van der Waals layered structure allows the preservation of crystal integrity throughout transfer, stacking, or thinning, enabling both bottom-up and top-down processing without rigid lattice-matching constraints.^20–22^ Beyond 2D materials, amorphous semiconductors offer another route toward flexible bottom-up and top-down processability, as their noncrystalline nature eliminates the requirement for lattice matching at the interface.^23,24^ Although amorphous semiconductors typically suffer from low carrier mobility and poor device performance, indium oxide (In_2_O_3_) is a notable exception.^25^ Even in its amorphous form, In_2_O_3_ delivers high field-effect mobilities exceeding 100 cm^2^ V^–1^ s^–1^, qualifying it as a rare high-performance amorphous semiconductor.^26–28^

This unique feature is advantageous in both bottom-up and top-down fabrication. For bottom-up processing, In_2_O_3_ can be deposited via ALD at low temperature, making it compatible with back-end-of-line (BEOL) thermal budgets and suitable for monolithic three-dimensional (3D) integration.^29,30^ For top-down processing, inductively coupled plasma and acid-based wet etching (HCl) are employed. However, while these methods effectively remove material, they often induce surface damage or offer limited thickness control.^31,32^ Therefore, developing a precise top-down technique capable of atomic-scale thinning while preserving surface smoothness remains challenging.

In this work, we present a robust top-down/bottom-up platform enabling repeated, atomic-scale dimensional control in the amorphous In_2_O_3_ material system. Leveraging the inherent chemical continuity of the amorphous surface, we developed a mild hydroxide-assisted wet-etching process using dilute NH_4_OH that achieves a low etch rate of ∼0.4 nm min^–1^ while maintaining surface roughness (*R*_a_) of 0.5 nm. This mild thinning preserves the surface chemical state, allowing subsequent low-temperature ALD regrowth and enabling bidirectional control of film thickness with subnanometer precision and uniformity. Notably, since film thickness directly governs the electrical properties of In_2_O_3_, this process facilitates doping-free modulation of the threshold voltage (*V*_T_) and allows for reversible switching between depletion- and enhancement-mode operation within the same device. Using precise channel thickness control, we successfully demonstrated In_2_O_3_ logic circuits, including inverters and multistage ring oscillators (ROs). This reversible thinning–regrowth platform establishes film thickness as a core design parameter for oxide semiconductors, offering a generalized route toward reconfigurable and monolithically integrated electronic systems.

Ultrathin In_2_O_3_ (<5 nm in thickness) has recently emerged as a promising channel material for advanced monolithic 3D integration. Reducing the thickness below 5 nm enables enhancement-mode operation in In_2_O_3_ transistors, achieving high on-currents exceeding 2 mA μm^–1^ at nanoscale gate lengths.^29,33,34^ To realize such high performance, maintaining a high surface smoothness is crucial to ensure consistent electrical properties and provide a defect-free interface for subsequent layer stacking.^26,35^ Concurrently, maintaining proper oxygen stoichiometry is essential, as oxygen vacancies and hydrogen-related defects strongly influence carrier density and device stability.^36,37^

For conventional deposition techniques, physical vapor deposition suffers from limited conformality,^38^ while chemical vapor deposition typically requires temperatures that exceed back-end thermal limits.^39^ In contrast, ALD addresses these challenges through self-limiting surface reactions, enabling precise thickness control and atomic-scale uniformity at low thermal budgets.^40,41^ In this study, thin film In_2_O_3_ is deposited by ALD using trimethylindium (TMI) and H_2_O at 275 °C (Figure 1a), which is well below the BEOL thermal budget of 350 °C. The ALD process ensures excellent conformality and achieves subnanometer roughness *R*_a_ = ∼0.5 nm, a prerequisite for advanced scaling (Figure S1). High-resolution transmission electron microscopy (TEM) image of the ALD-grown In_2_O_3_ film exhibit a disordered atomic arrangement, with no visible lattice fringes (Figure 1b). Complementary X-ray diffraction (XRD) analyses further confirm that the film is amorphous, as the spectra lack sharp crystalline peaks (Figure 1c).

**Figure 1:**
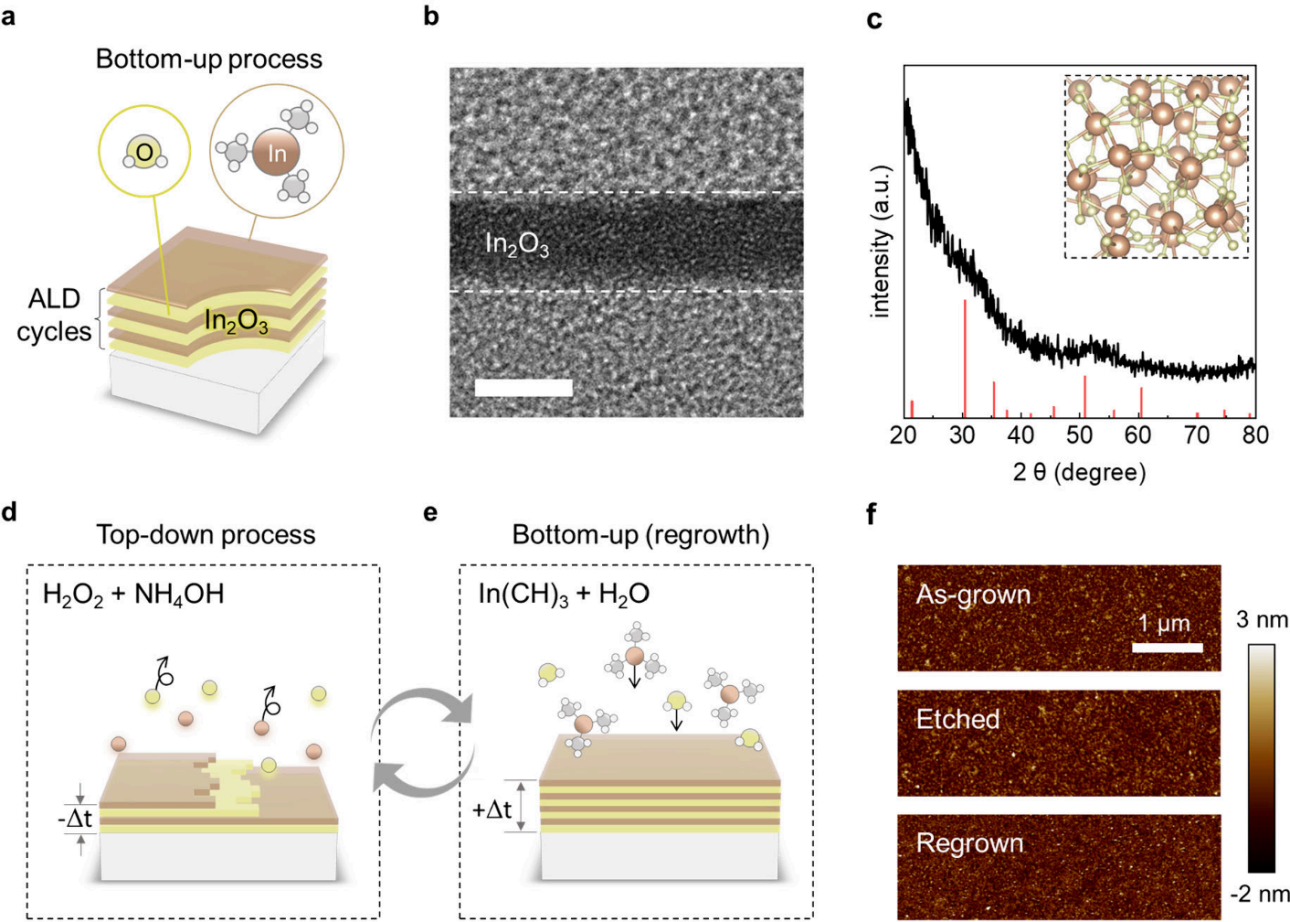
Schematic illustration of the top-down thinning and bottom-up regrowth cycle for amorphous In_2_O_3_. (a) ALD of In_2_O_3_ using TMI and H_2_O precursors. (b) TEM image of the as-deposited film. Scale bar: 3 nm. (c) XRD analysis of the In_2_O_3_ film. The inset shows a simulated structure highlighting its amorphous nature. (d) Atomic-scale wet-etching process enabling controlled top-down thinning. (e) ALD-based bottom-up regrowth of the thinned In_2_O_3_ layer. (f) AFM analyses for the as-grown, etched, and regrown films.

While the bottom-up growth of In_2_O_3_ can be precisely controlled by ALD, a top-down process capable of equally precise thickness control has yet to be developed. In conventional semiconductors such as Si and III–V compounds, thinning and patterning are typically achieved through anisotropic plasma etching or wet chemical etching.^42,43^ These processes rely on crystallographic directionality but require passivation to prevent sidewall damage and roughening, which degrade carrier transport and hinder subsequent deposition, as commonly observed in Si, GaN, and GaAs CMOS (complementary metal-oxide-semiconductor) fabrication.^44^

To achieve top-down atomic-scale thickness control in In_2_O_3_, we developed a mild wet-etching process that utilizes the surface chemistry of the amorphous In_2_O_3_ (Figure 1d). A dilute NH_4_OH (NH_4_OH:H_2_O_2_:H_2_O = 1:1:5) solution was employed to induce hydroxide-mediated dissolution of In_2_O_3_. In this solution, H_2_O_2_ facilitates surface hydroxylation to generate In–OH terminations, causing the In_2_O_3_ surface to exhibit chemical behavior similar to In­(OH)_3_.^45^ Under the alkaline environment provided by NH_4_OH, these hydroxylated surface species react with the OH^–^ ion to form soluble indium hydroxide complexes such as [In­(OH)_4_]^−^.^46^

The reaction proceeds slowly and uniformly, suppressing local overetching and maintaining a smooth surface morphology, thereby enabling a thinning step that can be subsequently reversed by ALD regrowth (Figure 1e) while preserving the same morphology roughness (Figure 1f). This slow dissolution gradually reduced the film thickness from 4 to 1.5 nm at a highly controlled rate of 0.33 ± 0.13 nm min^–1^, confirmed by atomic force microscopy (AFM) (Figures 2a and S2). Notably, this process achieves subnanometer precision, surpassing the monolayer limit of 2D semiconductors (e.g., ∼0.7 nm per layer for 2D transition-metal dichalcogenides). Due to the isotropic nature of amorphous In_2_O_3_, the film is etched uniformly by the mild dissolution process, maintaining morphological uniformity with *R*_a_ = ∼0.5 nm (Figure 2b). Due to the high surface uniformity after etching, the thickness of the In_2_O_3_ can be restored through ALD regrowth. The ALD process increases the thickness of etched In_2_O_3_ from 1.5 to 4 nm at a growth rate of 0.2 Å per ALD cycle (Figure 2c), and the regrown film maintains a surface roughness of *R*_a_ = ∼0.5 nm (Figure 2d). This alternating thinning and growth sequence enables reversible thickness tuning at the atomic scale, as shown in Figure 2e. The entire process exhibits uniformity in 1 cm^2^ area, supported by the optical micrographs in Figure 2f that show clear thickness-dependent contrast across both thinning and regrowth. Note that the proposed alternating thinning and regrowth sequence achieves true atomic-scale reversible thickness control that has not been demonstrated in other semiconductor systems. Although 2D materials support near-atomic thinning or regrowth, these processes are neither fully reversible nor scalable. The forgiving amorphous surface of In_2_O_3_ enables isotropic etching and defect-tolerant ALD regrowth, providing a uniquely reversible and low-temperature route for atomic-scale thickness tuning.

**Figure 2:**
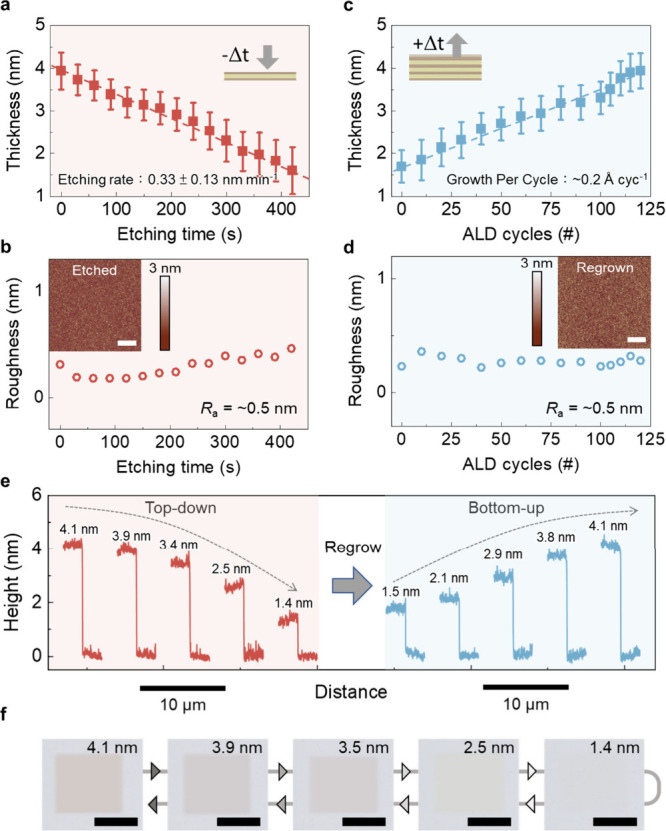
Thickness, surface morphology, and optical contrast of amorphous In_2_O_3_ during top-down thinning and bottom-up regrowth cycling. (a) Thickness of In_2_O_3_ reduced from 4 to 1.5 nm by a wet-etching process. (b) Surface roughness (*R*_a_) during the top-down process measured by AFM. Scale bars: 1 μm. (c) Thickness of In_2_O_3_ increased from 1.5 to 4 nm by an ALD process. (d) Surface roughness (*R*_a_) during the bottom-up process measured by AFM. Scale bars: 1 μm. (e) AFM step-height profiles acquired after sequential thinning and regrowth. (f) Optical micrographs of In_2_O_3_ films with different thicknesses (4.1, 3.9, 3.5, 2.5, and 1.4 nm). Scale bars: 20 μm.

Postetching regrowth is possible due to the chemically preserved surface, which maintains effective In–OH nucleation sites for ALD. X-ray photoelectron spectroscopy (XPS) was used to monitor the O 1s and In 3d core levels across the growth–thinning–regrowth processes. In the O 1s spectra (Figure 3a), the lattice-related peak at lower binding energy near 530 eV remains unchanged, while the peak near 531.5–532.5 eV,^47–49^ corresponding to the hydroxyl-associated component and the underlying SiO_2_, becomes more pronounced after thinning due to the reduced In_2_O_3_ thickness (XPS peak deconvolutions for etched and regrown films with various thicknesses are shown in Figure S3, and we also compare as-grown 1.4 nm film with etched film in Figure S4). The In 3d_5/2_ and In 3d_3/2_ spectra (Figure 3b) also remain constant, with binding energies at 444.6 and 452.2 eV, indicating that the indium oxidation state is unaffected.^50^ For the regrowth process, the preserved In–OH-terminated surface provides effective nucleation sites, enabling conformal regrowth without altering the local bonding environment. Consequently, the XPS shown in Figure 3c,d exhibits a trend and features identical to the initial state at the same thicknesses. This overall invariance of the surface chemistry demonstrates that amorphous In_2_O_3_ maintains chemical continuity throughout the entire thinning and regrowth cycle, thereby strongly supporting repeated top-down and bottom-up atomic-scale thickness modulation.

**Figure 3:**
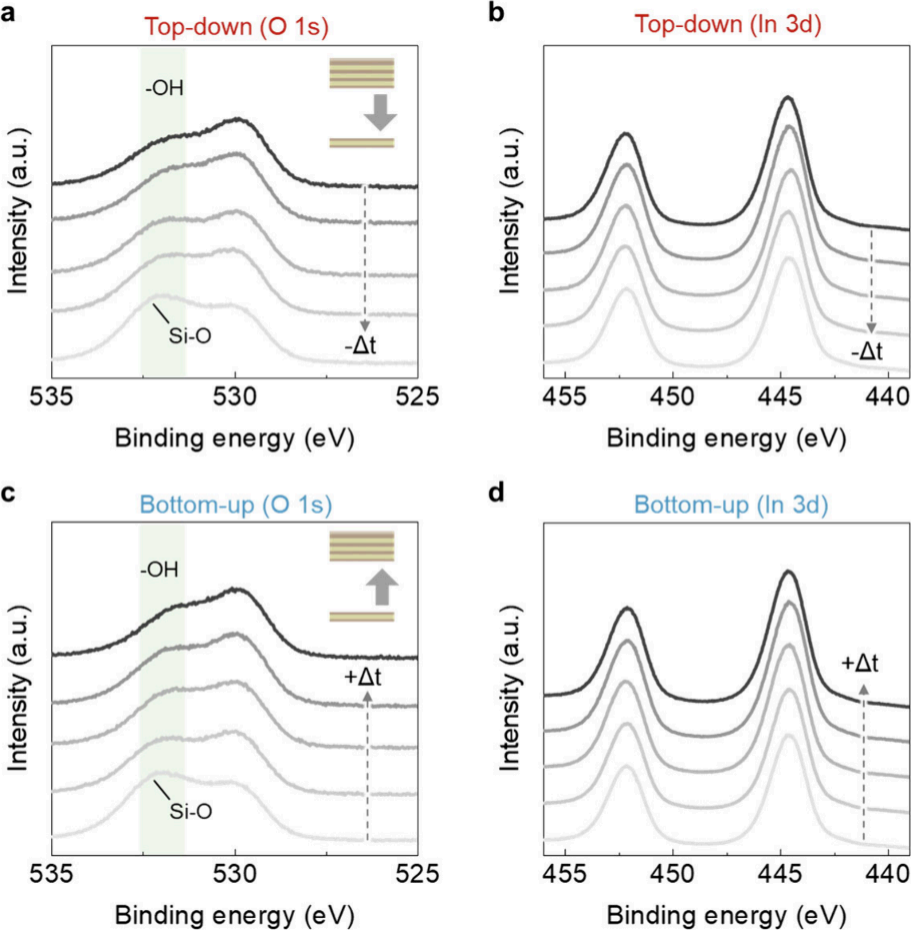
XPS of amorphous In_2_O_3_ before etching, after etching, and after regrowth. (a) O 1s spectra of the as-made and etched In_2_O_3_ films. (b) In 3d_5/2_ and In 3d_3/2_ spectra of the as-made and etched In_2_O_3_ films. (c) O 1s spectra of the regrown In_2_O_3_ films. (d) In 3d_5/2_ and In 3d_3/2_ spectra of the regrown In_2_O_3_ films.

The ability to precisely tune the In_2_O_3_ film thickness, as achieved by the proposed thinning–regrowth process, offers a powerful mechanism for controlling transistor performance. The electrical characteristics of In_2_O_3_ are strongly influenced by channel thickness, specifically the *V*_T_, which exhibits a sensitive dependence as the film thickness is reduced to the nanoscale.^26^ This thickness scaling effect was previously attributed to quantum confinement-induced band-gap widening with a pinned charge neutrality level.^30,51^ More recent studies show that the *V*_T_ shifts are associated with scaling effects in amorphous structures governed by percolation transport.^52^ Crucially, this tunability allows the *V*_T_ of In_2_O_3_ transistors to be adjusted across both depletion- and enhancement-mode operation regimes.

Electrical measurements were conducted on In_2_O_3_ transistors with a planar ultrathin-body structure, where the channel thickness was first reduced from 4 to 1 nm through atomic-scale wet etching and then regrown to 4 nm by ALD, as shown in Figure 4a. At a film thickness of 4 nm, the In_2_O_3_ transistor exhibits a degenerate channel that cannot be turned off. As the film thickness is gradually reduced by atomic-scale etching, the transfer characteristics (*I*_D_–*V*_G_ curve) shift progressively toward higher gate bias, indicating a transition from degenerate to depletion and finally to enhancement mode, as illustrated in Figure 4b. The extracted *V*_T_ and field-effect mobility, summarized in Figure 4c, show a positive *V*_T_ shift from −29.7 to +26.4 V and a concurrent decrease in mobility (Figure S5a), consistent with enhanced surface and interface scattering in the ultrathin regime. When In_2_O_3_ is regrown from 1 nm back to 4 nm (Figure 4d), the transfer characteristics shift back toward lower gate bias and recover the initial off-state behavior, as shown in Figure 4e. Correspondingly, Figure 4f shows *V*_T_ drifts back from approximately +20.5 to −29 V, and mobility increases along the same trajectory (Figure S5b). The *V*_T_ shows a positive shift as thickness is reduced during the thinning cycles and a negative shift as thickness is increased during regrowth cycles. This *V*_T_-thickness dependence is qualitatively consistent with both the quantum confinement and percolation transport models (discussion in the Supporting Information and Figure S6). Note that the *V*_T_ shifts for as-grown and etched In_2_O_3_ with various thicknesses are similar, showing that *V*_T_ correlates strongly with thickness, regardless of whether the film was etched or grown (Figure S7). The matched characteristics after etch-down and regrowth confirm negligible process damage and identify film thickness as the key reversible control of device electrostatics. Furthermore, stability characterizations were conducted as shown in Figures S8 and S9. The transfer characteristics exhibit negligible hysteresis and a low interface trap density (*N*_it_), indicating a high-quality dielectric/channel interface. Moreover, the *V*_T_ distribution demonstrates remarkable consistency across both individual samples and different batches, confirming the excellent uniformity and reproducibility of the hydroxide-assisted thinning process.

**Figure 4:**
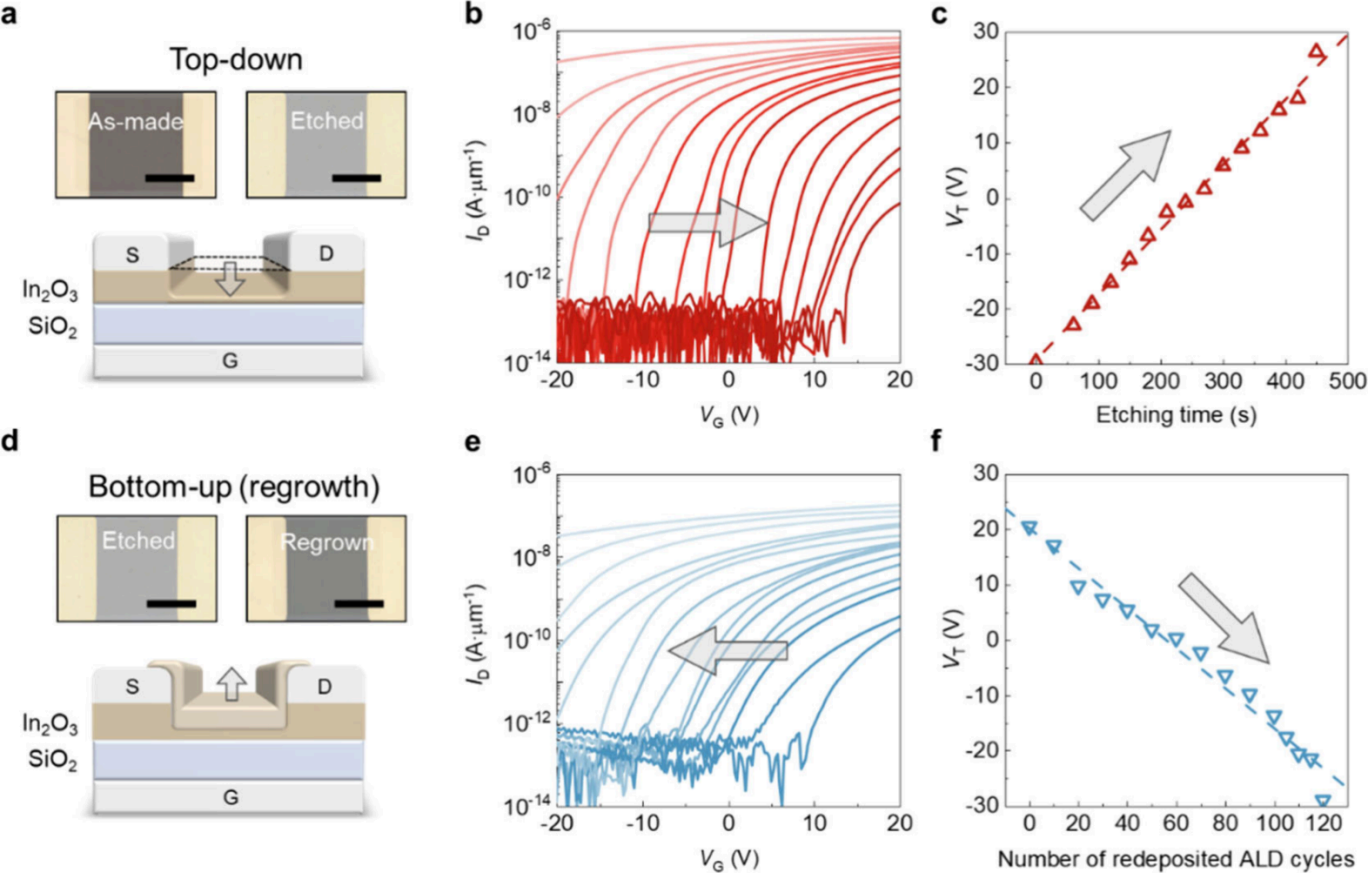
Electronic impact of thickness modulated by the thinning and regrowth processes on In_2_O_3_ transistors. (a) Optical micrographs of the as-made and etched devices and the corresponding schematic cross section during the thinning step. Scale bars: 25 μm. (b) Transfer characteristics of ultrathin In_2_O_3_ transistors with thicknesses ranging from 4 to 1.4 nm by the thinning process under *V*_DS_ = 0.1 V. (c) Change in *V*_T_ extracted from part b. (d) Optical micrographs of the etched and regrown devices and the corresponding schematic cross section during the regrowth step. Scale bars: 25 μm. (e) Transfer characteristics of ultrathin In_2_O_3_ transistors with thicknesses ranging from 1.5 to 4 nm redeposited by ALD under *V*_DS_ = 0.1 V. (f) Change in *V*_T_ extracted from part e.

In previous studies, *V*_T_ of In_2_O_3_ could be tuned by various doping, annealing, and surface engineering strategies. Controlled doping methods, including cation substitution (e.g., Sn or Ga incorporation) and anion modulation through oxygen partial pressure or plasma treatments, have been explored to tune *V*_T_ by compensating for native donors or introducing shallow acceptors.^24,53^ Postdeposition annealing, in either oxidizing or reducing ambient, further modifies defect states and carrier density, thereby shifting *V*_T_ through chemical equilibration of the oxygen vacancies.^54–56^ In addition, surface charge control via fluorine or self-assembled molecular doping provides additional flexibility for threshold tuning without altering the bulk film.^34^ In contrast to these approaches, the present thickness-modulated strategy provides a doping-free and reversible means to tune *V*_T_, where the channel thickness itself serves as a primary electrostatic control parameter for precise and uniform control across oxide semiconductor devices.

Using atomic-scale thickness modulation as a tuning parameter, a doping-free n-type metal-oxide-semiconductor (NMOS) inverter was realized, as shown in Figure 5a. The fabrication process sequentially involved the deposition of local gates on a quartz substrate, followed by a 10 nm HfO_2_ dielectric layer, 4 nm In_2_O_3_ channel layer, and source/drain/interconnect electrodes. An inverter is composed of two serially connected transistors, i.e., load transistor and driver transistor, as shown in the optical micrograph in Figure 5b. Initially, both transistors operated in depletion mode, and therefore the circuit exhibited poor inversion function, showing a weak voltage-transfer characteristic (VTC) and a low gain (Figure 5c). By selectively thinning the channel of the drive transistor via atomic-scale wet etching, the drive transistor turned into an enhancement mode, and the NMOS inverter then functioned properly through complementary threshold characteristics. The transfer characteristics shifted toward higher gate bias, corresponding to a positive shift in *V*_T_ from −1 to +1 V without degradation of the contacts or the dielectric (Figure S10).^57^ As a result, the VTC of the inverter displays a sharp transition, with *V*_OUT_ switching from *V*_DD_ to 0 V as *V*_IN_ exceeds the *V*_T_ of the drive transistor, as shown in Figure 5d. The voltage gain increases from 36 to 80 V as *V*_DD_ is raised from 2 to 5 V, confirming signal amplification. The transient response shown in Figure 5e further confirms stable dynamic operation, as a 4 V, 10 kHz square-wave input produces a clean inverted output waveform, verifying full inverter functionality enabled by doping-free threshold modulation simply through atomic-scale thickness control.

**Figure 5:**
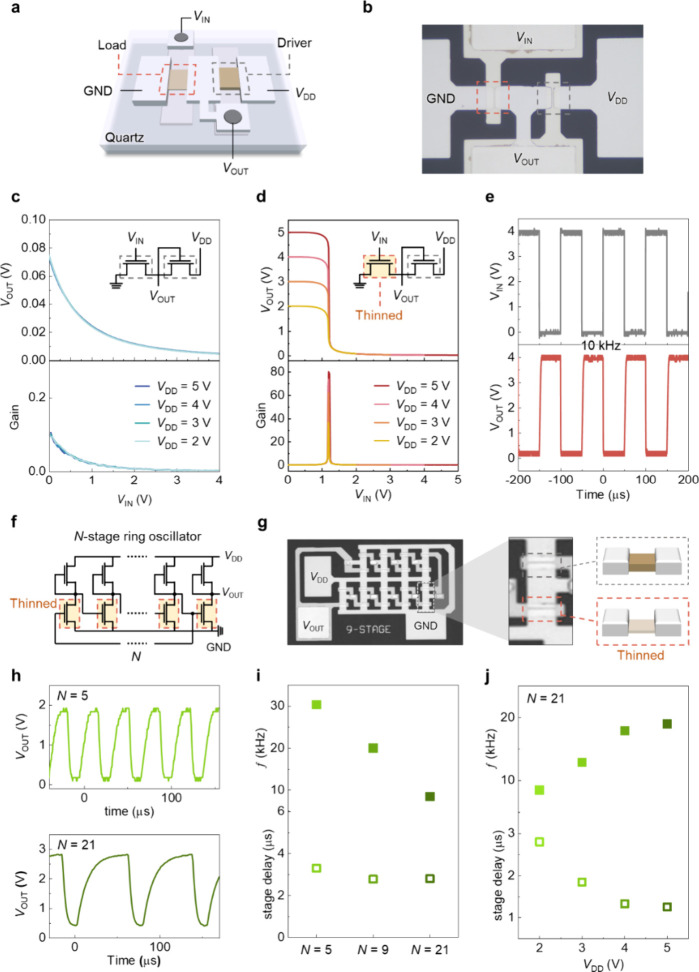
Demonstration of the NMOS inverter and RO using the atomic-scale thickness control processing technology. (a) Schematic of the NMOS inverter. (b) Optical micrograph of the NMOS inverter. (c) VTC and corresponding voltage gain of the inverter before etching process. (d) VTC and corresponding voltage gain of the inverter after selective thinning process. (e) Transient response of the inverter operating at a frequency of 10 kHz with *V*_DD_ set to 4 V. (f) Circuit diagram of the RO. (g) Optical micrograph of the RO. (h) Output waveforms of oscillators with different numbers of stages. (i) Frequency and stage-delay analysis of ROs with different stage counts at *V*_DD_ = 2 V. (j) Frequency and stage-delay analysis of the 21-stage ROs with *V*_DD_ ranging from 2 to 5 V.

Multiple inverters were further interconnected to form ROs to demonstrate the scalability and uniformity of the doping-free thickness-control approach. Figure 5f presents the circuit diagram of the RO, which consists of an odd number of inverters connected in a loop to enable oscillation. The RO was fabricated following the same process as the inverter, in which the circuit layout was first defined and the drive transistors in each stage were subsequently locally etched to enable oscillation, as shown in the optical micrograph in Figure 5g. The output waveforms of the ROs are shown in Figure 5h, all exhibiting voltage swings exceeding 1 V with stable signal propagation. As the number of stages increases from 5 to 21, the oscillation frequency decreases from 30.3 to 8.5 kHz (Figure 5i), while the per-stage delay remains nearly constant, indicating high device uniformity and reproducible atomic-scale thickness control. The dependence of oscillation frequency on supply voltage exhibits increased frequency and reduced delay time while increasing *V*_DD_ (Figure 5j), a behavior attributed to the reduced on-resistance (*R*_on_) of the constituent transistors at higher operating voltages. Furthermore, Figure S11 illustrates the results of negative bias stress (NBS) and positive bias stress (PBS) tests conducted on both the load and driver transistors, demonstrating that the inverter and oscillator circuits would remain stable under continuous operational stress. This consistent scaling behavior verifies robust circuit operation and highlights the potential of selective atomic-scale etching for large-area, doping-free oxide semiconductor integration.

This study develops an atomic-scale etching technique for amorphous In_2_O_3_ and integrates it with ALD regrowth to achieve precise, bidirectional, and low-damage thickness modulation, as confirmed by stable surface morphology (*R*_a_ = ∼0.5 nm) and XPS spectra. This integration realizes the repeated synergy of bottom-up and top-down processing within the same oxide system, enabling reversible and uniform thickness control in a BEOL-compatible manner. Such controllability allows *V*_T_ to be tuned continuously and device operation modes to be reversibly switched between depletion and enhancement modes on the same transistor, leading to doping-free inverters and multistage ROs with uniform stage delay and stable operation. This reversible atomic-scale dimensional engineering framework provides a conceptual and technological blueprint for realizing programmable functionality in a wide range of amorphous material systems.

## Supplementary Material



## Notes

Experimental fabrication details and characteristic data for In_2_O_3_ thin films and devices, including XPS and AFM analyses (PDF)
