# Supplementary material for: Reversible Thickness Engineering in Amorphous In2O3 Transistors
Source: Nano Lett. 2026 Apr 13;26(19):6270–7. doi: 10.1021/acs.nanolett.5c06228 (PMC13195641; doi:10.1021/acs.nanolett.5c06228)
Supplement: Supplementary file 1 [file nl5c06228_si_001.pdf]

Reversible Thickness Engineering in Amorphous In_2O_3 Transistors

*Yi-Yu Pan[1], Chu-Hsiu Hsu[2], Robert Tseng[1], Sung-Tsun Wang[2], Yu-Cheng Chang[1], Shih-Chieh Chen[2], Takashi Kimura[3], Yann-Wen Lan[4], Der-Hsien Lien[1]**

[1]Institute of Electronics, National Yang Ming Chiao Tung University, Hsinchu, Taiwan.

[2]Institute of Pioneer Semiconductor Innovation, National Yang Ming Chiao Tung University, Hsinchu, Taiwan.

[3]Department of Physics, Kyushu University, Fukuoka, Japan.

[4]Department of Physics, National Taiwan Normal University, Taipei, Taiwan.

*Address correspondence to dhlien@nycu.edu.tw

METHODS

1. ALD Growth and Top-Down Wet-Etch Thinning

Amorphous In_2O_3 films were grown in a home-built ALD system at 275 ℃ using TMI and H_2O as indium and oxygen precursors, respectively. Each ALD cycle consisted of alternating TMI and H_2O pulses separated by high-purity N_2 purges to remove residual species. To enhance nucleation on SiO_2 surfaces, ten initial H_2O-only pre-pulses were introduced prior to steady-state cycling. The growth proceeded with a growth per cycle (GPC) of approximately 0.2 Å $cycle^{-1}$, and the total film thickness was adjusted by the number of ALD cycles. Top-down thinning of the In_2O_3 film was performed by a mild hydroxide-assisted wet-etch process using a dilute NH_4OH solution ($NH_4OH:H_2O_2:H_2O$ = 1:1:5) at room temperature. Samples were rinsed in deionized (DI) water and cleaned with acetone and isopropanol before and after etching. For the experiments in Figure 2 and Figure 4, the thinning was carried out by immersing blanket films directly into the etchant without prior patterning. In contrast, for the integrated inverter and oscillator circuits presented in Figure 5, photolithography was first uesd to define the driver FETs, allowing for the selective etching of only the designated channel regions. The etch rate was calibrated on witness samples and found to vary linearly with etch time under fixed concentration and temperature, yielding an etching rate of approximately 0.4 nm min^{-1}. Film thickness was precisely controlled by adjusting immersion time, and the reaction was terminated by DI-water rinsing followed by N_2 drying. The process used short immersions and maintained compatibility with pre-patterned device topography.

2. Material Characterization

The In_2O_3 thickness was determined by AFM and TEM. AFM topography was measured using a Bruker Dimension Icon operated in contact mode at room temperature. Samples were solvent-cleaned in acetone immediately before scanning. Cross sections were

prepared by focused-ion-beam (FIB) lift-out and examined on a JEOL JEM-2010F operated at 200 kV. XPS of the In 3d and O 1s core levels was acquired on a home-built system with a Mg Kα source (hν = 1253.6 eV), and an electron flood gun was used for charge neutralization.

3. Transistor, Inverter, and Ring Oscillator Fabrication

For transistors on Si, heavily phosphorus-doped Si wafers with 30 nm SiO_2 served as substrates. A 4 nm In_2O_3 layer was deposited by ALD. Active areas were defined by photolithography and etched in dilute HCl ($HCl:H_2O$ = 1:50) for 10 s. Ni electrodes were deposited by electron-beam evaporation and patterned by lift-off. For inverters and ROs on quartz, a 30 nm Ni local back-gate was deposited by electron-beam evaporation, followed by ALD of 10 nm HfO_2 and 2.5 nm In_2O_3. Active areas were defined by stepper lithography and wet-etched in dilute HCl ($HCl:H_2O$ = 1:50) for 10 s. Contact vias were opened by stepper lithography and BCl_3/Ar plasma dry etching. Ni (30 nm) was then deposited by electron-beam evaporation and patterned by lift-off to form source/drain ohmic contacts and interconnects. Finally, the drive transistor active region was redefined by stepper lithography and thinned by top-down wet-etch thinning.

4. Transistor and Ring-Oscillator Electrical Characterization

For transistors, electrical measurements were performed on a probe station at room temperature using an Agilent B2902B source/measure unit. V_T of In_2O_3 transistors was extracted by the constant-current method at $I_D = 10\ \text{nA} \times W_{ch}/L_{ch}$. The field-effect mobility (μ) in the linear regime was calculated from the transconductance as $\mu = \frac{L_{ch}}{W_{ch}}\frac{1}{C_{ox}V_{DS}}(\frac{\partial I_D}{\partial V_G})$ evaluated at low V_{DS} = 0.1 V. For ROs, output waveforms were measured on a RIGOL MSO5074 mixed-signal oscilloscope. The oscillation frequency (f_{osc}) was obtained from the waveform period. The per-stage delay was computed as $\tau_{stage} = 1/(2N f_{osc})$ for an N-stage ring.

5. Estimation of the V_T–thickness dependence using quantum confinement and percolation transport

To interpret the threshold voltage variation after the top-down thinning and subsequent bottom-up regrowth processes, we evaluate the V_T–thickness dependence using two physical models: quantum confinement (QC) and percolation transport. The assumptions, derivations, and parameters used in each model are summarized below.

In the QC scenario, we assume that the threshold voltage corresponds to the gate bias required to raise the Fermi level (E_F) to a fixed energy separation below the conduction band minimum (E_C). When the channel thickness (t) decreases, E_C shifts upward due to confinement. This band-edge shift can be approximated using the infinite quantum well model, expressed as $\Delta E_C \approx \frac{\pi^2\hbar^2}{2m^*t^2}$, where $\hbar$ is the reduced Planck constant and m^* denotes the electron effective mass of amorphous In_2O_3. [1,2] To estimate how the gate field modifies E_F, we consider that an applied gate bias (ΔV_G) increases the electrostatic potential according to the partition between the oxide capacitance (C_{OX}) and the semiconductor capacitance (C_S). The resulting Fermi level shift is therefore written as $\Delta E_F = \frac{C_{OX}}{C_{OX}+C_S} q\Delta V_G$, where q is the elementary charge. [3] By equating the additional Fermi level shift to the confinement-induced ΔE_C, the threshold voltage (V_T) variation required for turn-on can be approximated as $\Delta V_T \approx \frac{\Delta(E_c - E_F)}{q}(1 + \frac{C_S}{C_{OX}})$, which directly leads to a thickness dependence approaching t^{-2}. As shown in **Figure S3**, this model predicts a rapidly increasing V_T as the channel approaches the ultrathin regime, resulting in a non-linear behavior that is steeper than the experimental trend. This mismatch suggests that although QC affects band edge alignment, it alone cannot explain the magnitude and slope of V_T variation.

In the percolation transport scenario, to describe the thickness-dependent transport behavior in disordered oxide semiconductors, we model the local potential landscape using a random

band-edge (mobility-edge) model.[4] Each spatial site is assigned a mobility-edge energy E_{M} drawn from a Gaussian distribution,

$$g(E_{\mathrm{M}}) = \frac{1}{\sigma\sqrt{2\pi}}\exp\left(-\frac{E_{\mathrm{M}}^2}{2\sigma^2}\right)$$

, where σ is the standard deviation of energy variations, characterizing the disorder strength of the system, representing long-range potential fluctuations inherent to amorphous oxides. This disordered potential landscape determines the spatial variation of accessible electronic states.

For each site, the total density of states is composed of extended states above $E_M$and exponentially-distributed tail states below it:

$$D(E,E_{\mathrm{M}}) = \begin{cases} D_{c0}\sqrt{(E-E_{\mathrm{M}})+(N_{\mathrm{m}}/D_{\mathrm{c0}})^2}, & E > E_{\mathrm{M}} \\ N_{\mathrm{m}}\exp\left[\dfrac{E-E_{\mathrm{M}}}{E_0}\right], & E < E_{\mathrm{M}} \end{cases}$$

, where $D(E, E_{\mathrm{M}})$ is the energy-dependent density of states (DOS), D_{C0} is the conduction band DOS prefactor, N_{m} is the density of localized states, and E_0 is the characteristic energy width of localized states. The local carrier density is then obtained by integrating the DOS weighted by the Fermi–Dirac distribution:

$$n(E_{\mathrm{M}},E_{\mathrm{F}}) = \int D(E,E_{\mathrm{M}})f(E,E_{\mathrm{F}})\,dE$$

, where $f(E, E_{\mathrm{F}})$ is the Fermi-Dirac distribution. Repeating this calculation over all randomly generated sites yields a spatial carrier-density distribution that evolves with the Fermi level. A site is considered conducting when

$$n(E_{\mathrm{M}},E_{\mathrm{F}}) > n_{\mathrm{th}}$$

, where $n_{\mathrm{th}}$is the critical 3D carrier density required for extended transport. The percolation probability $p(E_{\mathrm{F}})$is defined as the fraction of conducting sites.

To relate percolation behavior to measurable electrical characteristics, the average carrier density

$$n_{\mathrm{avg}}(E_{\mathrm{F}}) = \langle n(E_{\mathrm{M}}, E_{\mathrm{F}}) \rangle$$

is converted into an equivalent sheet density using a constant geometrical factor δ_z, and the corresponding gate voltage is obtained from the capacitive relation

$$V_G(E_F) = V_{\mathrm{ref}} + \frac{q\, n_{\mathrm{avg}}(E_F)\, \delta_z}{C_{\mathrm{ox}}}.$$

Combining these steps produces a direct mapping $p(V_G)$between percolation probability and gate voltage. By comparing this model-derived $p(V_G)$curve with experimental data, we determine how the observed threshold behavior originates from thickness-dependent percolation rather than quantum-confinement-induced band-edge shifts.

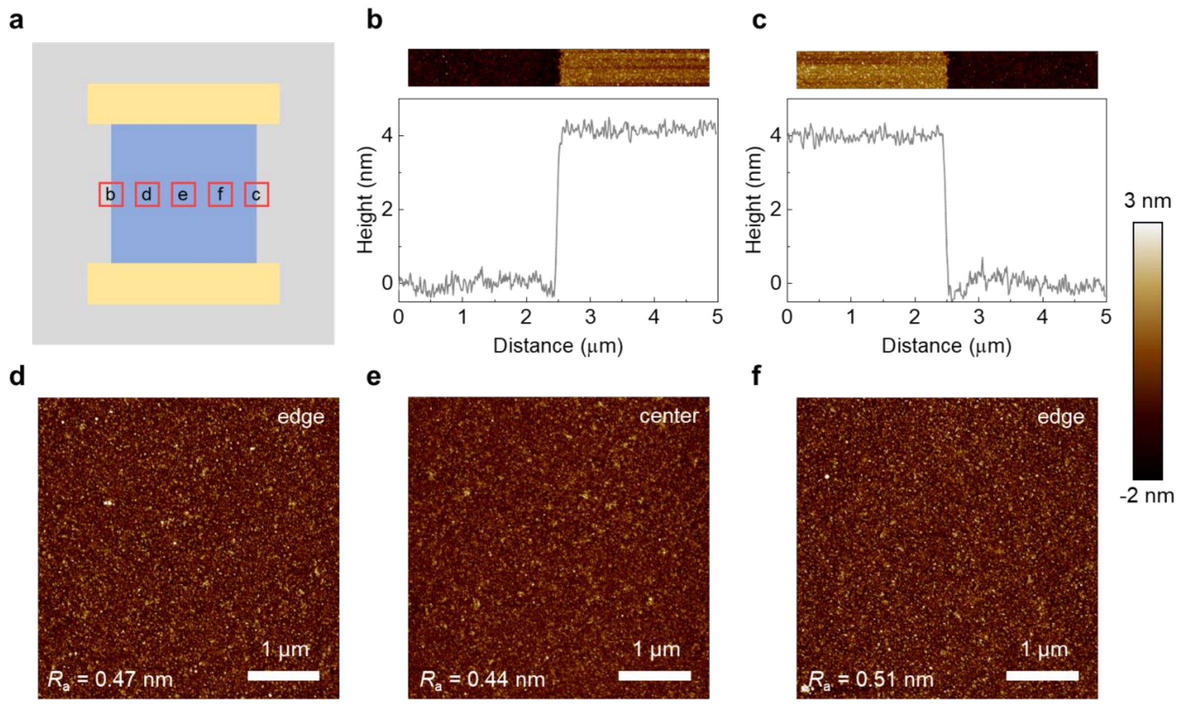

Figure S1. AFM analysis of ALD-grown In_2O_3. (a) The selected area of the In_2O_3 for AFM analyses. (b) The height analysis at the left edge. (c) The height analysis at the right edge. (d) The roughness analysis near left edge. (e) The roughness analysis at center. (f) The roughness analysis near the right edge.

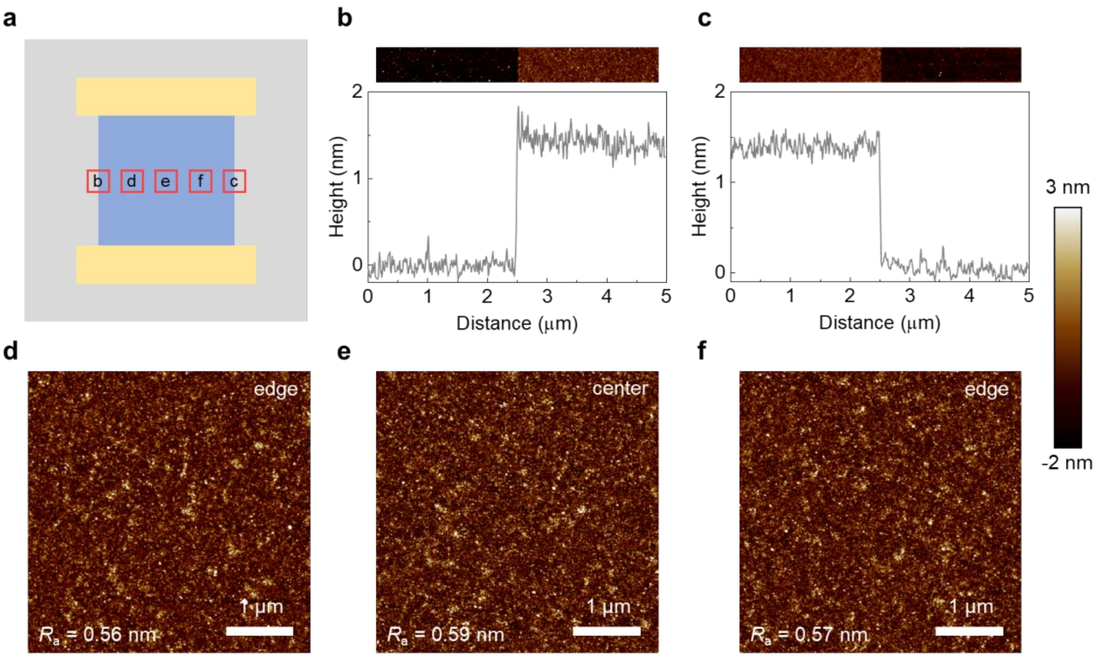

Figure S2. AFM analysis of etched In_2O_3. (a) The selected area of the In_2O_3 for AFM analyses. (b) The height analysis at the left edge. (c) The height analysis at the right edge. (d) The roughness analysis near left edge. (e) The roughness analysis at center. (f) The roughness analysis near the right edge.

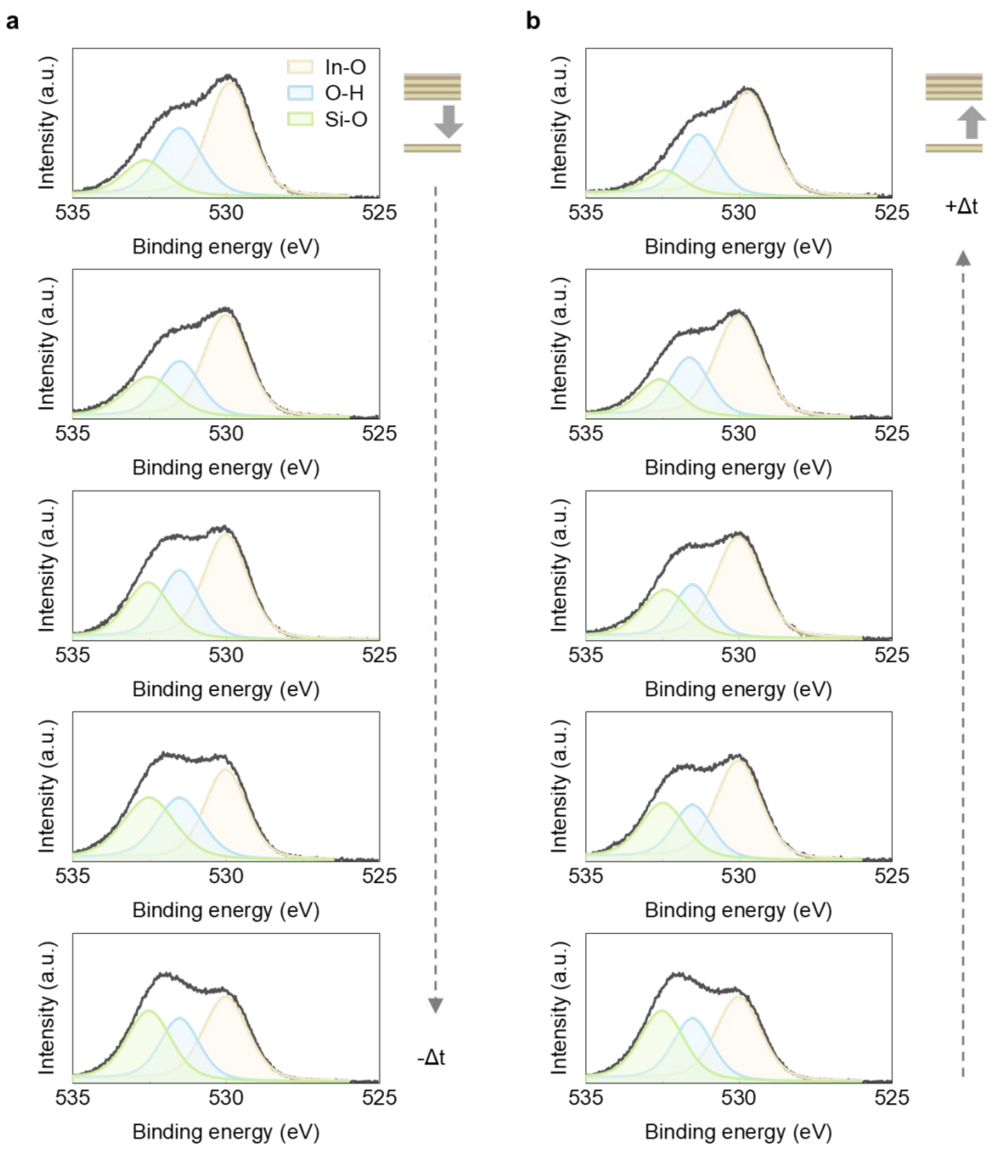

Figure S3. XPS spectra of the (a) etched In_2O_3 films with gradually reducing thicknesses (top to bottom) and (b) regrown In_2O_3 films with gradually increasing thicknesses (bottom to top). For thinner films (whether etched or regrown), the In-O signals are lower than the Si-O signals. This occurs because as the channel thickness is reduced, the Si-O signal from the underlying SiO_2 becomes more pronounced in the spectrum, given that the XPS probing depth is typically a few nanometers. For thicker films, the results are reversed, with the In-O signal becoming stronger than the Si-O signal as the channel material occupies a greater volume of the sampling depth.

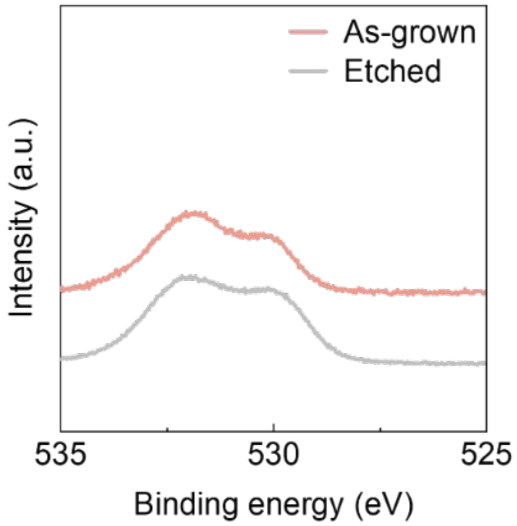

Figure S4. XPS spectra of an etched In_2O_3 film and an as-grown (unetched) film, both with a thickness of 1.4 nm film. There is no significant difference between the etched and as-grown films of the same thickness. This confirms that the increased intensity at higher binding energy is a thickness-related effect rather than an artifact of the chemical etching process.

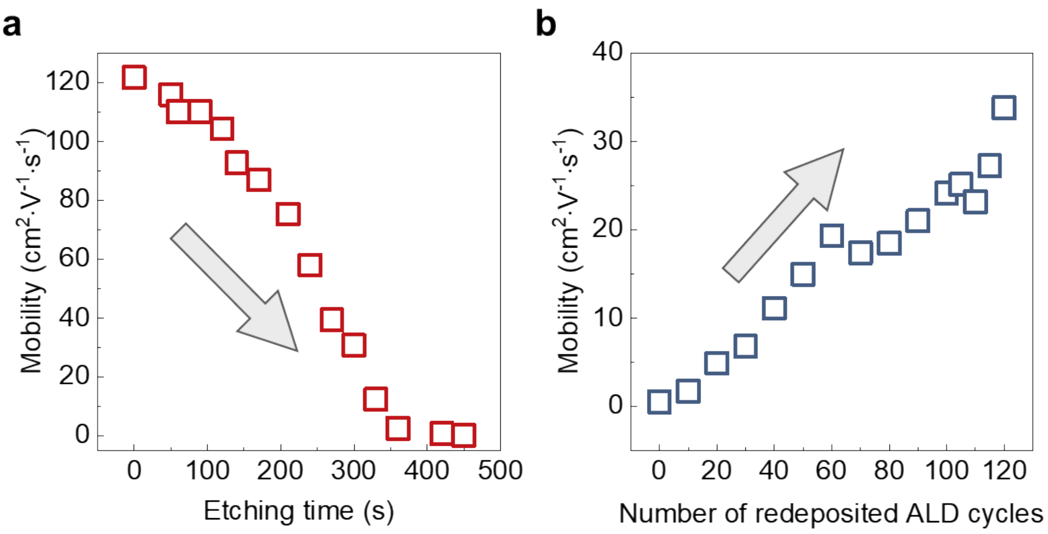

Figure S5. The change in mobility during the thinning and regrowth processes. (a) Mobility evolution during the top-down thinning process. (b) Mobility evolution during the bottom-up regrowth process.

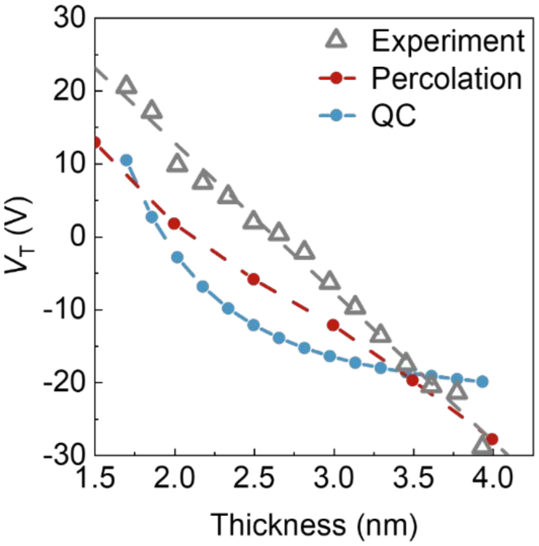

Figure S6. V_T as a function of channel thickness, showing experimental data, quantum confinement estimation, and percolation-based simulation.

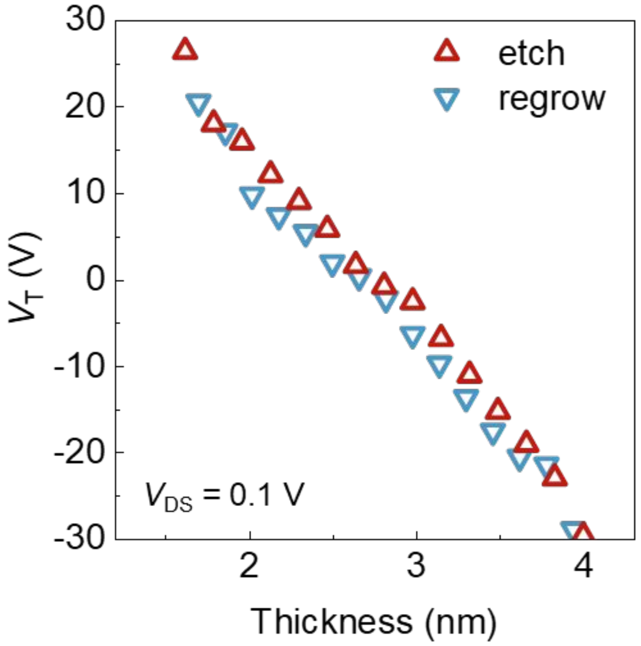

Figure S7. V_T shifts for regrown and etched In_2O_3 with various thicknesses.

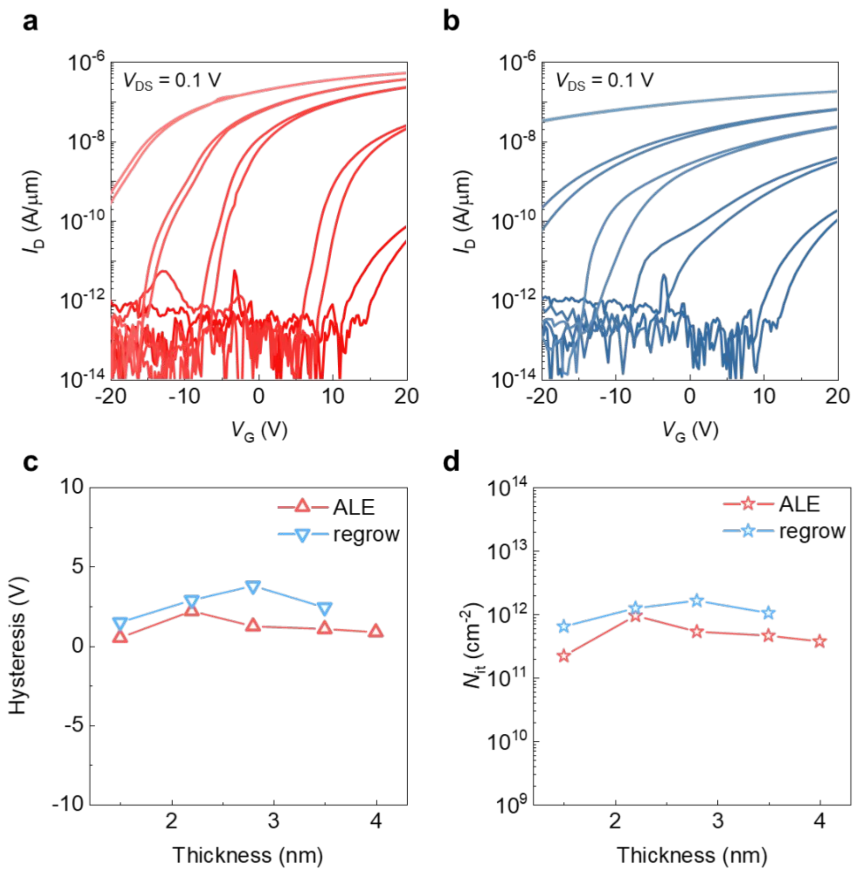

Figure S8. The transfer curves include both forward and backward gate voltage sweeps for (a) In_2O_3 transistors with thinning down processes and (b) In_2O_3 transistors with regrown processes. (c) The hysteresis windows extracted from transfer curves. (d) N_{it} extracted from the hysteresis.

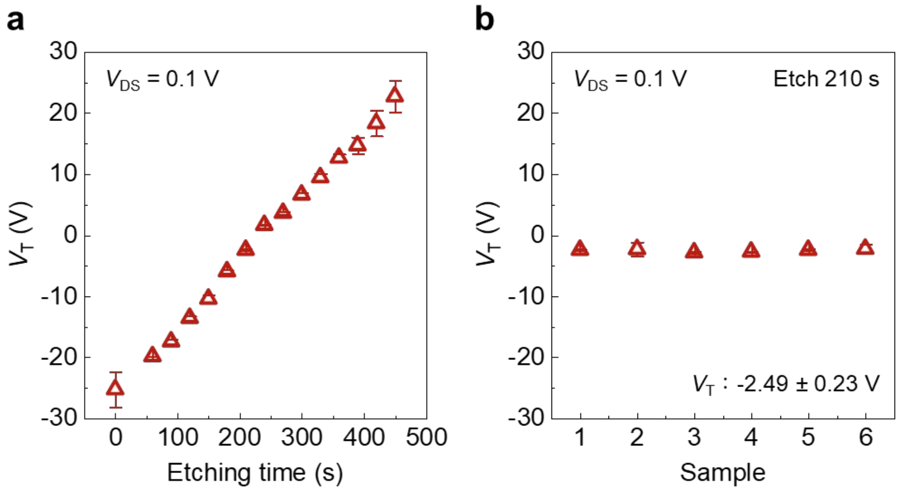

Figure S9. V_T variation across devices and chips. (a) The corresponding V_T as a function of etching time. (b) The corresponding V_T after 210 s of etching across 6 different chips.

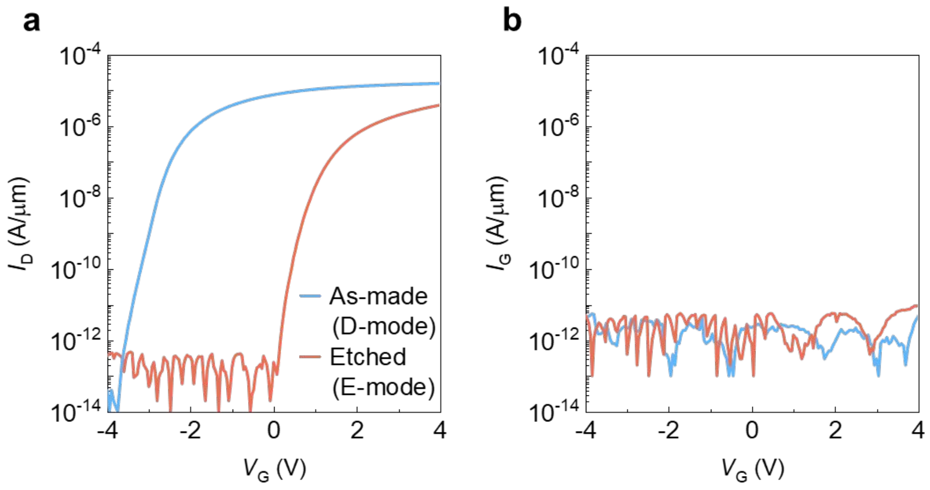

Figure S10. Electronic characteristics of the transistor before and after selective etching under V_{DS} = 0.1 V. (a) I_D-V_G curve. (b) I_G-V_G curves. From the contact resistance (R_c) analysis by I_D-V_G curve, we observed no significant difference before (361 Ω·μm) and after (667 Ω·μm) the thinning process. Additionally, the gate leakage (I_G) remained lower than the measurement limit.

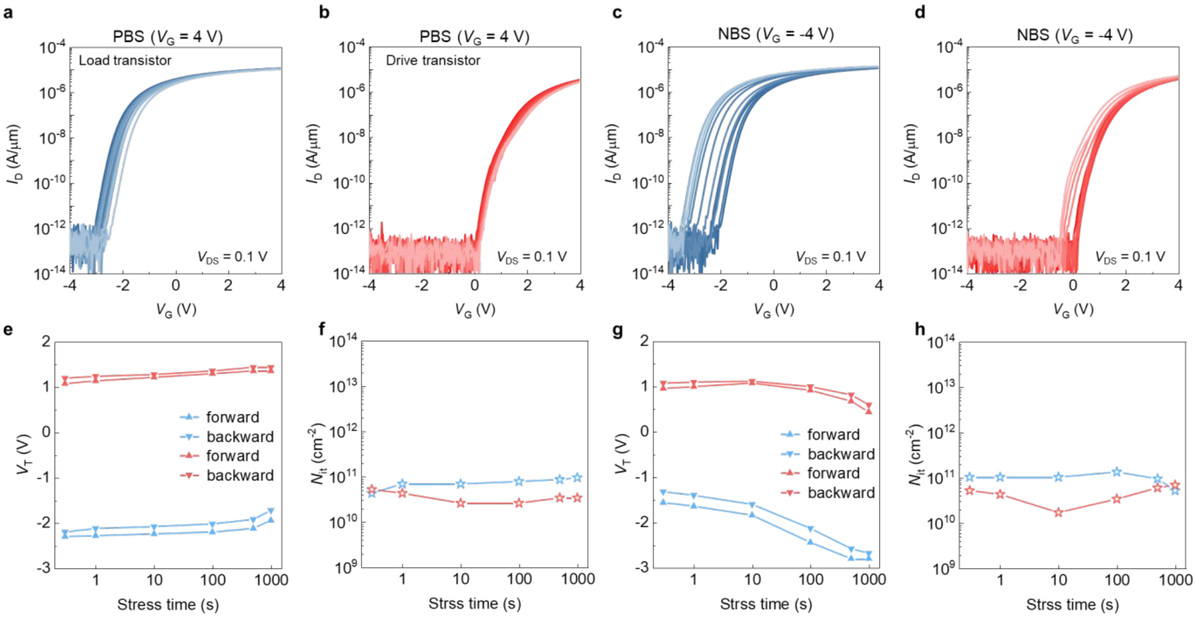

Figure S11. The drift of the transfer curves for (a) the load transistor and (b) the drive transistor under a PBS of 4 V. The drift of the transfer curves for (c) the load transistor and (d) the drive transistor under a NBS of -4 V. The corrseponding (e) V_T and (f) N_{it} drift as a function of stress time for the load transistor and the drive transistor under the PBS. The corresponding (g) V_T and (h) N_{it} drift as a function of stress time for the load transistor and the drive transistor under the NBS.

REFERENCE

(1) Si, M.; Hu, Y.; Lin, Z.; Sun, X.; Charnas, A.; Zheng, D.; Lyu, X.; Wang, H.; Cho, K.; Ye, P. D. Why In2O3 can make 0.7 nm atomic layer thin transistors. *Nano Lett.* **2020**, *21* (1), 500-506.

(2) Kumar, M. J.; Singh, T. V. Quantum Confinement Effects in Strained Silicon MOSFETs. *Int. J. Nanosci.* **2008**, *7*, 81-84.

(3) Tsutsui, G.; Saitoh, M.; Nagumo, T.; Hiramoto, T. Impact of SOI thickness fluctuation on threshold voltage variation in ultra-thin body SOI MOSFETs. *IEEE Trans. Nanotechnol.* **2005**, *4* (3), 369-373.

(4) Fishchuk, I. I.; Kadashchuk, A.; Bhoolokam, A.; de Jamblinne de Meux, A.; Pourtois, G.; Gavrilyuk, M.; Köhler, A.; Bässler, H.; Heremans, P.; Genoe, J. Interplay between hopping and band transport in high-mobility disordered semiconductors at large carrier concentrations: The case of the amorphous oxide InGaZnO. *Phys. Rev. B* **2016**, *93* (19), 195204.
